# Supplementary material for: Vibrio parahaemolyticus quorum sensing controls phage VP882 transmission
Source: mBio. 2026 May 19;17(6):e00737-26. doi: 10.1128/mbio.00737-26 (PMC13251360; doi:10.1128/mbio.00737-26)
Supplement: Supplemental Information — Supplemental figures and tables. [file mbio.00737-26-s0001.pdf]

SUPPLEMENTARY MATERIAL

***Vibrio parahaemolyticus* quorum sensing controls phage VP882 transmission**

Molly R. Sargen and Bonnie L. Bassler

Figure S1: Phage VP882 only infects *V. parahaemolyticus* with the O3:K6 K-antigen

Figure S2: VqmA-VqmR quorum sensing does not affect phage VP882 infection

Figure S3: *VPA1602-VPA1604* are controlled by the LuxO quorum-sensing system

Figure S4: Phage VP882 genome recombination occurs during superlysogenization

Table S1: Transposon insertion sites identified in the screen for the phage VP882 receptor

Table S2: Transposon insertion sites identified in the screen for the factor that prevents phage VP882 adsorption to *V. parahaemolyticus*

Table S3: Strains used in this study

Table S4: Oligonucleotides used in this study

Table S5: Plasmids used in this study

Supplemental References

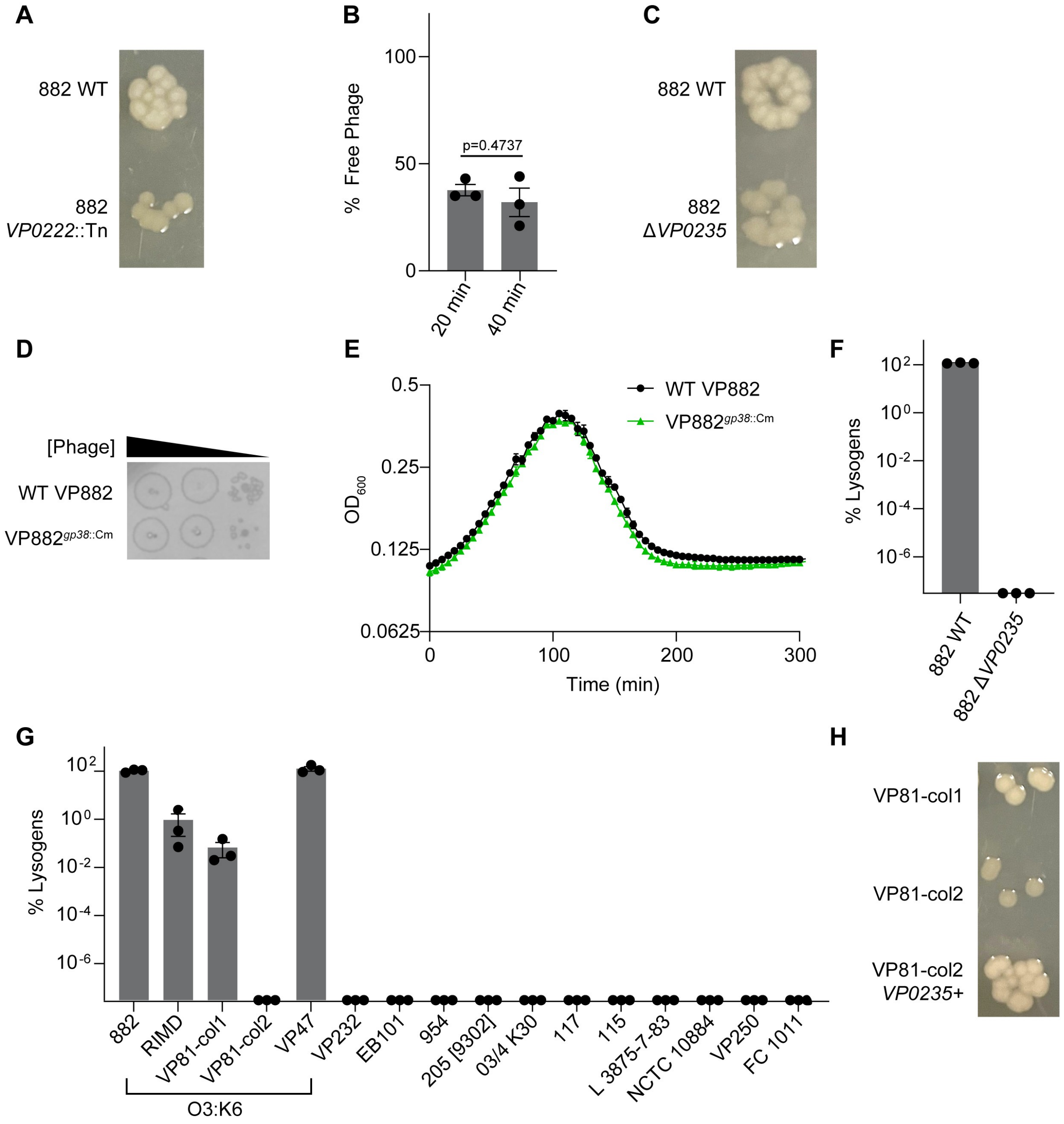

Figure S1: Phage VP882 only infects *V. parahaemolyticus* with the O3:K6 K-antigen

A) Colony morphologies of the designated phage-free *V. parahaemolyticus* 882 strains grown on LB agar at 37 °C for 16 h. B) Quantitation of phage VP882 adsorption to *V. parahaemolyticus* 882 cured of phage VP882 as in Fig. 1B except that free phage were sampled after 20 and 40 min of incubation. C) Colony morphologies of the designated phage-free strains as in A. D) Plaque formation by phage VP882 and phage VP882$^{gp38::Cm}$. Ten-fold serial dilutions of phage lysates (starting at 10^{-8}-10^{-9} PFU/mL) were spotted onto lawns of *V. parahaemolyticus* 882 that had been cured of phage VP882 embedded in 0.5% LB top agar overlaid onto 1.5% LB agar. Plaques were quantified after 16-20 h of incubation at 37 °C. E) Growth curves of *V. parahaemolyticus* 882

carrying phage VP882 or phage VP882$^{gp38::Cm}$ treated with mitomycin C (0.05 µg/mL). A representative of 2 biological replicates is shown. Each assay included 2 technical replicates. F) Quantitation of phage VP882$^{gp38::Cm}$ lysogens following infection of the designated phage-free strains (MOI 10^{-5}-10^{-6}). Lysogenization was carried out for 16 h. G) Quantitation of phage VP882$^{gp38::Cm}$ lysogens as in F. H) Colony morphologies of the designated phage-free strains as in A. In A, C, D, and H, a representative image from 3 biological replicates is shown. In B, F, and G, data are presented as means of 3 biological replicates. All error bars indicate SEMs. We note that in panel E, error bars are obscured by the symbols.

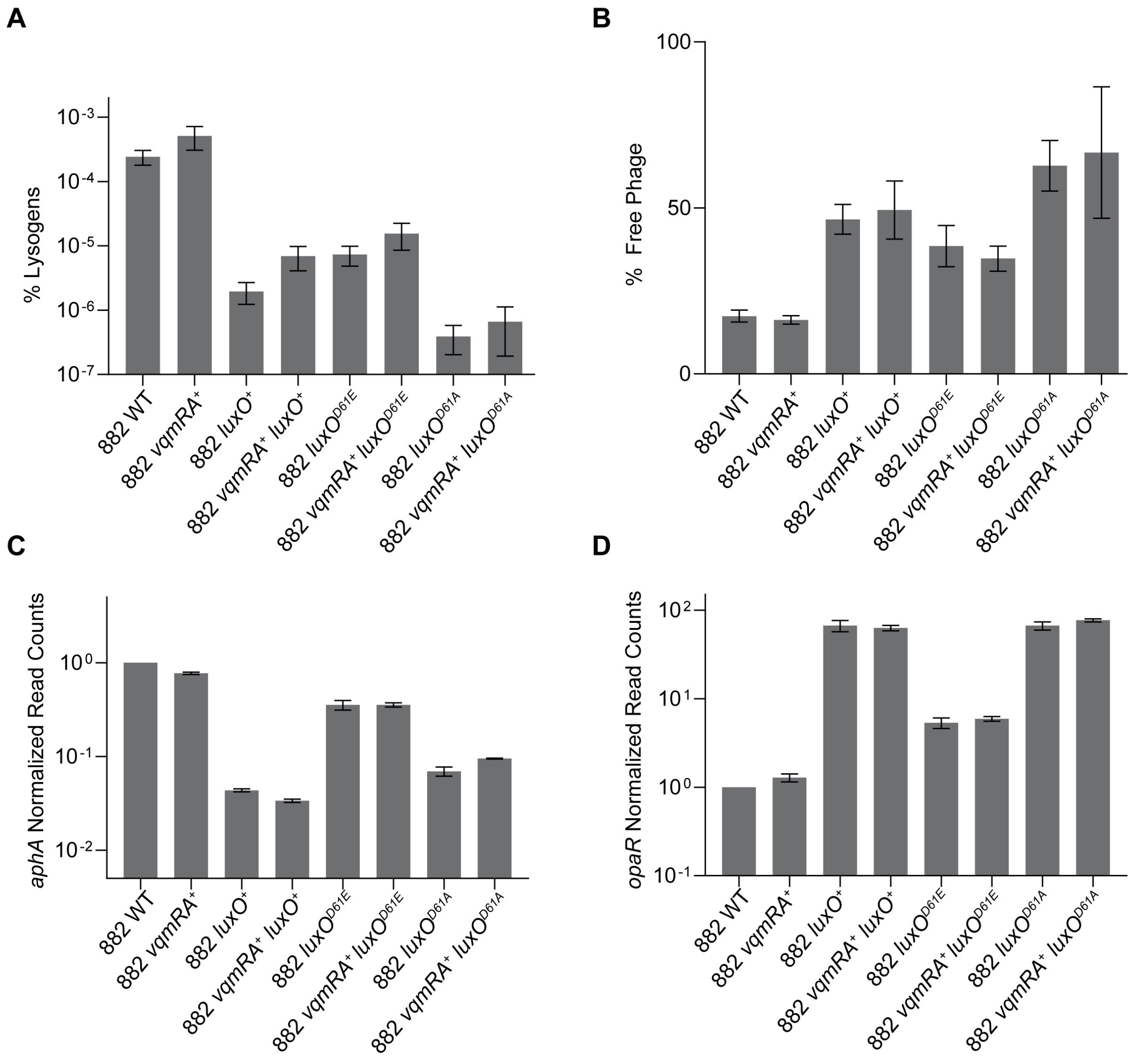

Figure S2: VqmA-VqmR quorum sensing does not affect phage VP882 infection

A) Quantitation of phage VP882 lysogens as in Fig. 3E. B) Quantitation of phage VP882 adsorption as in Fig. 2B. Data are presented as means of 3 biological replicates and error bars indicate SEMs. C) Quantitation of RNA-seq reads mapping to the *V. parahaemolyticus* master QS regulator *aphA* (*VP2762*) for strains with the indicated QS genotypes after 4 h of growth in LM at 30 °C (1). The fraction of total reads aligning to each gene were normalized to the corresponding value for *V. parahaemolyticus* 882. D) Quantitation of RNA-seq reads mapping to the *V. parahaemolyticus* master QS regulator *opaR* (*VP2516*) as in panel C.

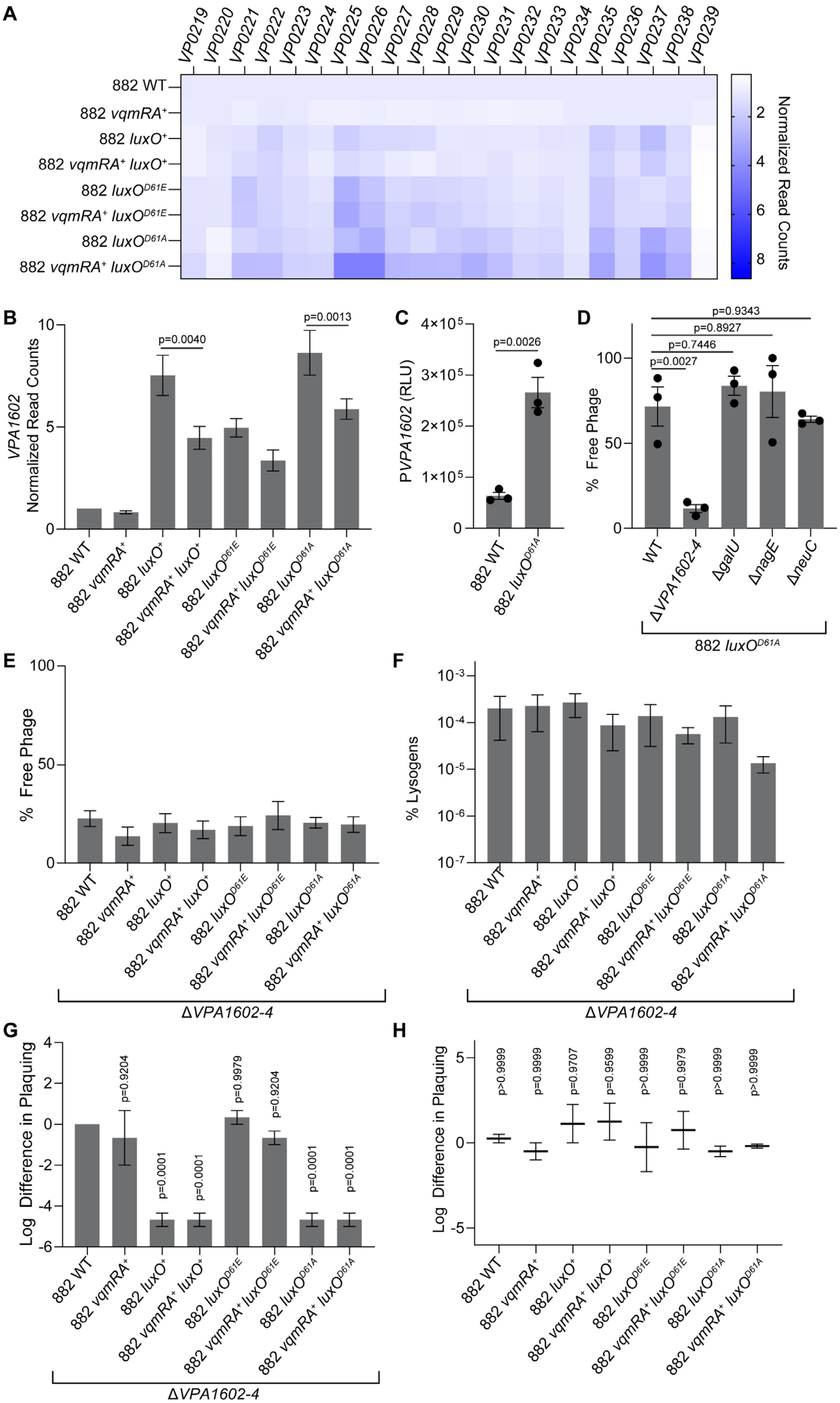

A
VP0219
VP0220
VP0221
VP0222
VP0223
VP0224
VP0225
VP0226
VP0227
VP0228
VP0229
VP0230
VP0231
VP0232
VP0233
VP0234
VP0235
VP0236
VP0237
VP0238
VP0239
882 WT
882 vqmRA+
882 luxO+
882 vqmRA+ luxO+
882 luxOD61E
882 vqmRA+ luxOD61E
882 luxOD61A
882 vqmRA+ luxOD61A
Normalized Read Counts
2
4
6
8
B
VPA1602
Normalized Read Counts
p=0.0040
p=0.0013
882 WT
882 vqmRA+
882 luxO+
882 vqmRA+ luxO+
882 luxOD61E
882 vqmRA+ luxOD61E
882 luxOD61A
882 vqmRA+ luxOD61A
C
PVPA1602 (RLU)
p=0.0026
4×10^5
3×10^5
2×10^5
1×10^5
882 WT
882 luxOD61A
D
% Free Phage
p=0.9343
p=0.8927
p=0.7446
p=0.0027
WT
ΔVPA1602-4
ΔgalU
ΔnagE
ΔneuC
882 luxOD61A
E
% Free Phage
ΔVPA1602-4
F
% Lysogens
10^-3
10^-4
10^-5
10^-6
10^-7
ΔVPA1602-4
G
Log Difference in Plaquing
p=0.9204
p=0.0001
p=0.0001
p=0.9979
p=0.9204
p=0.0001
p=0.0001
ΔVPA1602-4
H
Log Difference in Plaquing
p>0.9999
p>0.9999
p=0.9707
p=0.9599
p>0.9999
p=0.9979
p>0.9999
p>0.9999

Figure S3: *VPA1602-VPA1604* are controlled by the LuxO quorum-sensing system

A) Heatmap showing quantitation of RNA-seq reads mapping to the *V. parahaemolyticus* K-antigen locus for strains with the indicated QS genotypes under the conditions described in Fig. S2C. B) Quantitation of RNA-seq reads mapping to *VPA1602* under the conditions described in Fig. S2C. C) P*VPA1602-luxCDABE* output as in Fig. 3C except measurements were taken after 500 min and are reported on a linear scale. D and E) Quantitation of phage VP882 adsorption as in Fig. 2B. F) Quantitation of phage VP882 lysogens as in Fig. 3E. G) Quantification of phage VP882 plaque formation as in Fig. 3B. Note that, as designated, in panels E-G, *VPA1602-4* were deleted from all the strains. In panels E-G, each strain is compared to the parent *V. parahaemolyticus* 882 strain lacking *VPA1602-4* (leftmost bar in each case). H) Quantification of the differences in phage VP882 plaque formation scores on the indicated *V. parahaemolyticus* 882 strains containing or lacking *VPA1602-4*. For D-H, data are presented as means of 3 biological replicates and error bars indicate SEMs. In B and C, significance was determined by t-test to establish p-values for the indicated comparisons. In D, G, and H, significance was determined by one-way ANOVA with Tukey's test for multiple comparisons to establish adjusted p-values.

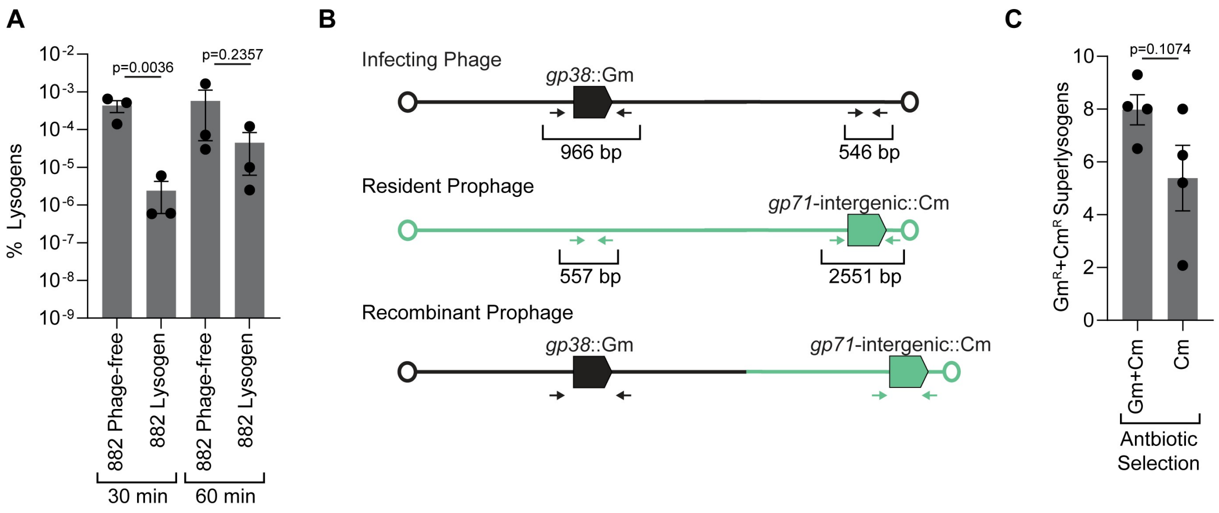

Figure S4: Phage VP882 genome recombination occurs during superlysogenization

A) Quantitation of phage VP882 lysogens at the indicated times as in Fig. 3E. B) Diagram of phage VP882 genomes used to monitor recombination in the superinfection experiments reported in Fig. 4D and F. C) Quantitation of doubly antibiotic resistant VP882 superlysogens selected on gentamicin + chloramphenicol or on only gentamicin. Colonies selected initially on medium containing only gentamicin were patched onto plates containing chloramphenicol to assess chloramphenicol resistance. In A and C, data are presented as means of 3 and 4 biological replicates, respectively, and error bars indicate SEMs. Significance was determined by t-test to establish p-values for the indicated comparisons.

Table S1: Transposon insertion sites identified in the screen for the phage VP882 receptor

Sequence Number	Insertion Site	Direction	Gene ID
1	231439	forward	*VP0220*
2	234489	forward	*VP0221*
3	234489	forward	*VP0221*
4	235609	forward	*VP0222*
5	235779	forward	*VP0222*
6	235855	forward	*VP0222*
7	235855	forward	*VP0222*
8	237039	forward	*VP0224*
9	243061	forward	*VP0230*
10	243498	reverse	*VP0231*
11	243643	forward	*VP0230*
12	243914	forward	*VP0230*
13	244073	forward	*VP0230*
14	244219	forward	*VP0231*
15	244285	forward	*VP0231*
16	244285	forward	*VP0231*
17	244479	forward	*VP0231*
18	249145	forward	*VP0235*
19	249360	reverse	*VP0235*

Table S2: Transposon insertion sites identified in the screen for the factor that prevents phage VP882 adsorption to *V. parahaemolyticus*

Insertions in *VPA1602-4* are shaded gray

* indicates the read quality was too poor to identify the insertion site

Sequence Number	Insertion Site	Direction	Disrupted locus	Annotation
Chromosome 1				
1	77696	reverse	*VP0068*	*gorA* glutathione-disulfide reductase
2	92662	reverse	*VP0081*	BON Domain protein
3	183123	forward	*VP0168-VP0170*, intergenic	TonB-dependent receptor plug domain-containing protein and ABC transporter
4	102615	reverse	*VP0109*	DMT transporter
5	128809	reverse	*VP0120*	*glnL* nitrogen regulation protein NR(II)
6	196839	reverse	*VP0183*	Methyl accepting chemotaxis protein
7	*	forward	*VP0199*	*neuC* UDP-N-acetylglucosamine 2-epimerase
8	209620	forward	5' *VP0199*	5' of *neuC* UDP-N-acetylglucosamine 2-epimerase
9	210461	forward	*VP0199*	*neuC* UDP-N-acetylglucosamine 2-epimerase
10	253049	reverse	*VP0238*	MBL fold metallo-hydrolase
11	379167	forward,	5' *VP0377*	HTH domain protein
12	390779	forward	3' *VP0388*	methylase
13	426994	forward	*VP0424*	*hldE* bifunctional D-glycero-beta-D-manno-heptose-7-phosphate kinase/D-glycero-beta-D-manno-heptose 1-phosphate adenylyltransferase
14	508389	forward	*VP0494*	*thrA* bifunctional aspartate kinase/homoserine dehydrogenase I
15	545647	reverse	*VP0527*	*nhaR* transcriptional activator
16	630725	forward	*VP0605*	rRNA large subunit methyltransferase N
17	681151	reverse	*VP06050, VP06051* intergenic	*nadK* NAD+ kinase*, grpE* nucleotide exchange factor
18	*	forward	*VP0682*	*ribH* 6,7-dimethyl-8-ribityllumazine synthase
19	716483	forward	*VP0684*	*thiL* thiamine-phosphate kinase
20	827688	forward	5' *VP0793*	*crr* PTS glucose transporter subunit IIA
21	*	reverse	*VP0831*	*nagE* N-acetylglucosamine-specific PTS transporter subunit IIBC
22	1022658	reverse	*VP0980*	DUF2062 domain protein
23	*	forward	*VP1231*	*fabV* enoyl-ACP reductase

24	1386862	reverse	*VP1305/VP1306*	*cobU,* adenosylcobinamide-GDP ribazoletransferase
25	1421362	reverse	*VP1340*	collagenase
26	2292011	reverse	3' *VP2183*	response regulator
27	2549724	forward	*VP2429-VP2430,* intergenic	*radA* DNA repair protein
28	2643101	forward	*VP2504*	*pcnB* polynucleotide adenylyltransferase
29	2688526	forward	5' *VP2546*	*csrA* carbon storage regulator
30	2688526	forward	5' *VP2546*	*csrA* carbon storage regulator
31	2723599	forward	*VP2577*	*rseA* sigma-E factor negative regulatory protein
32	2723599	forward	*VP2577*	*rseA* sigma-E factor negative regulatory protein
33	2755274	reverse	*VP2611*	*gshB* glutathione synthase
34	2833881	forward	*VP2683*	PhoH family protein
35	2867011	forward	*VP2711*	*galU* UTP--glucose-1-phosphate uridylyltransferase
36	2971496	reverse	*VP2807*	*rnr* ribonuclease R
37	3055472	forward	*VP2884*	*dusB* tRNA dihydrouridine synthase
38	3056590	forward	*VP2885*	*fis* DNA-binding transcriptional regulator
39	3056590	forward	*VP2885*	*fis* DNA-binding transcriptional regulator
40	3070488	reverse	*VPr018*	23S rRNA
41	3100523	forward	*VP2911*	5' of *hupA* nucleoid-associated protein HU-alpha
42	3220203	reverse	*VP3016*	HvfC/BufC N-terminal domain-containing protein
43	3247914	forward	*VP3041*	LysM domain protein
Chromosome 2				
44	37139	forward	*VPA0045*	Ada regulatory protein
45	44076	reverse	*VPA0063*	Hypothetical protein
46	452925	reverse	*VPA0495*	AraC family transcriptional regulator
47	*	forward	*VPA0627*	5' of *cyoA* ubiquinol oxidase subunit II
48	*	forward	*VPA1442*	DUF11 domain protein
48	1560611	forward	*VPA1464*	Lcl domain protein
49	1700868	forward	*VPA1602*	Polysaccharide export protein Wza
50	1700867	forward	*VPA1602*	Polysaccharide export protein Wza
51	1701292	reverse	*VPA1602*	Polysaccharide export protein Wza
52	1700403	forward	5' *VPA1602*	5' of *VPA1602* Polysaccharide export protein Wza
53	1799976	forward	*VPA1678*	*araC* transcriptional regulator
54	2932536	reverse	*VPA2764*	Homoserine dehydrogenase

Table S3: Strains used in this study

Identifier	Genotype	Plasmid/Phage	Source
V. parahaemolyticus 882			
MRS19		cured	This study, courtesy of O. Duddy
MRS21		VP882$^{gp71\text{-intergenic::Cm}}$	Duddy et al. 2023 (1)
MRS34		VP882$^{\text{ctr::Tn5}}$	Silpe and Bassler 2019 (2)
MRS70		VP882 (native)	Lan et al. 2009 (3)
MRS138		Cured; pMRS7	This study
MRS187	*vqmR*$^{+}$-P *vqmA*$_{882}$- *vqmA*$_{882}$:: *vqmA*$_{882}$ (*vqmR-vqmA*882^{+})	cured	This study
MRS188	*luxO*882::*luxO*RIMD (*luxO*$^{+}$)	cured	This study
MRS189	*vqmR*$^{+}$-P*vqmA*$_{882}$- *vqmA*$_{882}$:: *vqmA*$_{882}$; *luxO*882::*luxO*$_{RIMD}$ (*vqmR-vqmA*882^{+} *luxO*$^{+}$)	cured	This study
MRS312	*luxO*$_{882}$::*luxO*^{D61E} (*luxO*^{D61E})	cured	This study
MRS313	*vqmR*$^{+}$-P*vqmA*$_{882}$-*vqmA*$_{882}$::*vqmA*$_{882}$; *luxO*$_{882}$::*luxO*^{D61E} (*vqmR-vqmA*$_{882}$$^{+}$ *luxO*^{D61E})	cured	This study
MRS314	*luxO*$_{882}$::*luxO*^{D61A} (*luxO*^{D61A})	cured	This study
MRS417	*vqmR*$^{+}$-P*vqmA*$_{882}$-*vqmA*$_{882}$::*vqmA*$_{882}$; *luxO*$_{882}$::*luxO*^{D61A} (*vqmR-vqmA*$_{882}$$^{+}$ *luxO*^{D61A})	cured	This study
MRS541		VP882 $^{gp38\text{::Cm}}$	This study
MRS542		VP882 $^{gp38\text{::Gm}}$	This study
MRS550	Δ*VPA1602-1604*	cured	This study
MRS551	*luxO*$_{882}$::*luxO*^{D61A} (*luxO*^{D61A}) Δ*galU*	cured	This study
MRS552	*luxO*$_{882}$::*luxO*^{D61A} (*luxO*^{D61A}) Δ*nagE*	cured	This study
MRS553	*luxO*$_{882}$::*luxO*^{D61A} (*luxO*^{D61A}) Δ*neuC*	cured	This study
MRS554	*luxO*$_{882}$::*luxO*^{D61A} (*luxO*^{D61A}) Δ*VPA1602-1604*	cured	This study
MRS566	*vqmR*$^{+}$-P *vqmA*$_{882}$-*vqmA*882:: *vqmA*$_{882}$ (*vqmR- vqmA*$_{882}$$^{+}$) Δ*VPA1602-1604*	cured	This study
MRS567	*luxO*882::*luxO*$_{RIMD}$ (*luxO*$^{+}$) Δ*VPA1602-1604*	cured	This study
MRS568	*luxO*$_{882}$::*luxO*^{D61E} (*luxO*^{D61E}) Δ*VPA1602-1604*	cured	This study
MRS569	*vqmR*$^{+}$-P*vqmA*$_{882}$-*vqmA*$_{882}$::*vqmA*$_{882}$; *luxO*$_{882}$::*luxO*^{D61E} (*vqmR-vqmA*$_{882}$$^{+}$ *luxO*^{D61E}) Δ*VPA1602-1604*	cured	This study
MRS571	*vqmR*$^{+}$-P*vqmA*$_{882}$-*vqmA*$_{882}$::*vqmA*$_{882}$; *luxO*$_{882}$::*luxO*^{D61A} (*vqmR-vqmA*$_{882}$$^{+}$ *luxO*^{D61A}) Δ*VPA1602-1604*		This study
MRS574	*vqmR*$^{+}$-P*vqmA*$_{882}$-*vqmA*$_{882}$::*vqmA*$_{882}$; *luxO*882::*luxO*$_{RIMD}$ (*vqmR- vqmA*$_{882}$$^{+}$ *luxO*$^{+}$) Δ*VPA1602-1604*		
MRS703	Δ*VPA1602-1604*	VP882 (native)	This study
MRS707	*luxO*$_{882}$::*luxO*^{D61A} (*luxO*^{D61A}) Δ*VPA1602-1604*	VP882 (native)	This study
MRS759	Δ*VP0230*	cured	This study
MRS769		VP882 (native); FJS-P073	This study
MRS770	*luxO*882::*luxO*$_{RIMD}$ (*luxO*$^{+}$)	VP882 (native); FJS-P073	This study
MRS806		cured; pMRS70	This study

MRS812	$luxO_{882}$::$luxO^{D61A}$ ($luxO^{D61A}$)	cured; pMRS70	This study
MRS956	$luxO_{882}$::$luxO^{D61A}$ ($luxO^{D61A}$) Δ*VP0235*		This study
MRS958	Δ*VPA1602-4* Δ*VP0235*		This study
MRS959	$luxO_{882}$::$luxO^{D61A}$ ($luxO^{D61A}$) Δ*VPA1602-1604* Δ*VP0235*		This study
MRS970	Δ*recA*	cured	This study
MRS976	Δ*recA*	VP882 (native)	This study
MRS1011	Δ*recA*	VP882$^{gp71\text{-intergenic::Cm}}$	This study
V. parahaemolyticus VP81			
MRS729	col1		ATCC
MRS730	col2		ATCC
MRS758	col2 $VP0235^{+}$		This study
V. parahaemolyticus VP47			
MRS743			ATCC
MRS777	$luxO^{+}$		This study
MRS775		FJS-P073	This study
MRS778	$luxO^{+}$	FJS-P073	This study
Other *V. parahaemolyticus*			
MRS8	*V. parahaemolyticus* RIMD2210633		Makino et al.(4)
MRS733	*V. parahaemolyticus* VP232		ATCC
MRS734	*V. parahaemolyticus* EB101		ATCC
MRS735	*V. parahaemolyticus* 954		ATCC
MRS736	*V. parahaemolyticus* 205		ATCC
MRS737	*V. parahaemolyticus* 03/4 K30		ATCC
MRS738	*V. parahaemolyticus* 117		ATCC
MRS739	*V. parahaemolyticus* 115		ATCC
MRS740	*V. parahaemolyticus* MDL 3875-7-83		ATCC
MRS741	*V. parahaemolyticus* 10884		ATCC
MRS742	*V. parahaemolyticus* VP250		ATCC
MRS744	*V. parahaemolyticus* FC 1011		ATCC
E. coli strains for cloning and conjugative donors			
E. coli MFDλpir	MG1655 RP4-2-Tc::[ΔMu1::*aac(3)IV*-Δ*aphA*-Δ*nic*35 ΔMu2::*zeo*] Δ*dapA*::(*erm-pir*) Δ*recA*		Ferrières et al. 2010 (5) Obtained from the S. Helaine Lab.
E. coli S17λ*pir*	Δ*lacU169 (ΦlacZΔM15) recA1 endA1 hsdR17 thi-1 gyrA96 relA1 λpir*		Simon et al. 1983 (6)
E. coli TOP10	F- *mcrA* Δ(*mrr-hsdRMS-mcrBC*) Φ80*lacZ*Δ*M15* Δ*lacX74 recA1 araD139* Δ(*ara leu*)*7697 galU galK rpsL* (Str^{R}) *endA1 nupG*		Invitrogen
E. coli pir2^{+}	F^{-} *Δlac*169 *rpo*S(am) *rob*A1 *cre*C510 *hsd*R514 *end*A *rec*A1 *uidA(ΔMlu*I)::*pir*		Invitrogen

Table S4: Oligonucleotides used in this study

Identifier	Name	Sequence (5'-3')	Purpose	Source
oMRS1	*qtip*-exR	CCGAAGAAAGCGCAGAAGC	check for presence of phage VP882	This study
oMRS2	VP882-*vqmA*-exR	TGCAATTTGTCGAGAAGGATGC	check for presence of phage VP882	This study
oMRS29	pRE1128-pTOXconvert-F	GTTTAAACCAGGTTAATTAATTT GAGAAGCGGTGTAAGTGAACTG	transfer pRE112 insert to pTOX	This study
oMRS30	pRE1128-pTOXconvert-R	CTCGAGACGTCCCGGGATGCATTT TCTTCTAGAGGTACCGCATGC	transfer pRE112 insert to pTOX	This study
oMRS138	Arb1D	GGCCAGGCCTGCAGATGATGNNNNN NNNNNGTAT	Mariner transposon insertion site identification	Truong et al. 2020 (7)
oMRS139	Arb2	GGCCAGGCCTGCAGATGATG	Mariner transposon insertion site identification	Truong et al. 2020 (7)
oMRS140	Neo-F	CGTTGGCTACCCGTGATATT	Mariner transposon insertion site identification	Truong et al. 2020 (7)
oMRS141	pSC189-1st-3	GGCTGACCGCTTCCTCGTGCTTTAC	Mariner transposon insertion site identification	Truong et al. 2020 (7)
oMRS142	*vqmA*host-outer_fwd	ACGCATTTCCTGTACCAGCA	check *vqmRA* of Vpara	This study, courtesy of T.V.P. Nguyen
oMRS143	*vqmA*host-outer_rev	AAGCGATTGGTTTGTTGCGT	check *vqmRA* of Vpara	This study, courtesy of T.V.P. Nguyen
oMRS194	d*VP0235*_downR	TCTTCTAGAGGTACCGCATGC GAATCCAGCTCATTAAAGTATGC	construction of pTOX plasmid for deletion	This study
oMRS195	d*VP0235*_ovF	ATATGGCAACTTGACGCTATACAATTT ATGAGTCAATC	construction of pTOX plasmid for deletion	This study
oMRS196	d*VP0235*_ovR	ATAGCGTCAAGTTGCCATATAAATTGT AGACGTTGC	construction of pTOX plasmid for deletion	This study
oMRS197	d*VP0235*_upF	GTAAGTGAACTGCATGAATTCCC GGAATCGATACACGCAGTACTG	construction of pTOX plasmid for deletion	This study
oMRS198	*VP0235*-ex-F	ACGGGAAGTATTGTTGTTAGCTC	check deletion	This study
oMRS199	*VP0235*-ex-R	GACTTCCACAACAAGTAGAATAACC	check deletion	This study
oMRS225	*0230*_downR	CTCGAGACGTCCCGGGATGCATTTAC ACTACAACTGCCATCATAAGG	construction of pTOX plasmid for deletion	This study
oMRS226	*0230*_upF	GTTTAAACCAGGTTAATTAATTT TTTCTGCATATTCGGTTTGGTTCG	construction of pTOX plasmid for deletion	This study
oMRS231	d*0230*_ovR	TCACCGCCTCAAAGTACTGATGAAGG TATAAAAC	construction of pTOX plasmid for deletion	This study
oMRS232	d*0230*_ovF	TCAGTACTTTGAGGCGGTGAGTGATG AAAAG	construction of pTOX plasmid for deletion	This study
oMRS233	*luxO*vph-ex-F2	GAGGGGTCGCTAATATATCAG	check *luxO* sequence	This study
oMRS27	oMS714	CTGCGCCATCAGATCCTTGG	check insert in pTOX	Sargen and Helaine 2024 (8)
oMRS28	oMS715	CGATATCTTCGAACCGGTGC	check insert in pTOX	Sargen and Helaine 2024 (8)
oMRS72	*luxO*vph-ex-R	TCAAATACATTAAGCGCTCTGAACC	check genotype	This study
oMRS310	pEVS*lux*_RBS_vectorF	GCTTAACTAAGTAACTAGTACAGG	PCR of pEVS-lux backbone	This study
oMRS311	pEVS*lux*_vector_R	TTAATTAACTCGAGCGGTACC	PCR of pEVS-lux backbone	This study

oMRS409	*gp38*_gm_ovR	GAGAGTAGGGAACTGCCAGGTAGGATCTCCGTTTACCAGTTATAAAT	construction of gentamicin marked *gp38* phage	This study
oMRS410	*gp38*_gm_ovF	ACCCAAGTACCGCCACCTAAAAAAGCCCGCACAAGGCGG	construction of gentamicin marked *gp38* phage	This study
oMRS411	gm_cassette_R	TTAGGTGGCGGTACTTGGGTC	construction of gentamicin marked *gp38* phage	This study
oMRS412	gm_cassette_F	CCTGGCAGTTCCCTACTC	construction of gentamicin marked gp38 phage	This study
oMRS445	*galU*_upF	GTTTAAACCAGGTTAATTAATTTTCTGCAGATTATGTTGTGATTGC	construction of pTOX plasmid for deletion	This study
oMRS446	*galU*_downR	CTCGAGACGTCCCGGGATGCATTTCACTTGGTAAACAATGTTATCTGC	construction of pTOX plasmid for deletion	This study
oMRS447	d*galU*_ovR	GCCTGAACAATTTTAAATTCACCAACTGTAAAAAAGG	construction of pTOX plasmid for deletion	This study
oMRS448	d*galU*_ovF	GAATTTAAAATTGTTCAGGCTCGTTACCAATG	construction of pTOX plasmid for deletion	This study
oMRS449	*nagE*_upF	GTTTAAACCAGGTTAATTAATTTTCAACGCTATGAATACCTTTTTTCG	construction of pTOX plasmid for deletion	This study
oMRS450	*nagE*_downR	CTCGAGACGTCCCGGGATGCATTTGGCTGAACTCGTATTGATGAGG	construction of pTOX plasmid for deletion	This study
oMRS451	d*nagE*_ovR	TTCGATGTAACTTAAGTTCCCCCTATAGGATTC	construction of pTOX plasmid for deletion	This study
oMRS452	d*nagE*_ovF	GGAACTTAAGTTACATCGAACTAATATTTGATTAGAGAC	construction of pTOX plasmid for deletion	This study
oMRS453	*neuC*_upF	GTTTAAACCAGGTTAATTAATTTTACGTAATGGAAACGATTGAGAG	construction of pTOX plasmid for deletion	This study
oMRS454	*neuC*_downR	CTCGAGACGTCCCGGGATGCATTTCCAGAGCCAGGACGTTTAATCG	construction of pTOX plasmid for deletion	This study
oMRS455	d*neuC*_ovR	AATCAGGTTCAACCGCGACCTTTTTCTGTGTC	construction of pTOX plasmid for deletion	This study
oMRS456	d*neuC*_ovF	GGTCGCGGTTGAACCTGATTTAAATAAGAAAGTAGAG	construction of pTOX plasmid for deletion	This study
oMRS457	*VPA16024*_upF	GTTTAAACCAGGTTAATTAATTTGCTCAAAACTGCTTACTGGCAG	construction of pTOX plasmid for deletion	This study
oMRS458	*VPA16024*_downR	CTCGAGACGTCCCGGGATGCATTTATGGCGAAATTATTGCAAGTTGATTTTG	construction of pTOX plasmid for deletion	This study
oMRS459	d*VPA16024*_ovR	CTAGATTAATGTTTTTTCATTCATCTGACGTAAAGAG	construction of pTOX plasmid for deletion	This study
oMRS460	d*VPA16024*_ovF	ATGAAAAAACATTAATCTAGATCGCTAATTTGACC	construction of pTOX plasmid for deletion	This study
oTN0155	P*cat*-*cat*_fwd	TGATCGGCACGTAAGAGGTTCCAACTTTC	construction of phages marked at *gp38*	This study, courtesy of T.V.P. Nguyen
oTN0156	P*cat*-*cat*_rev	TTACGCCCCGCCCTGCCA	construction of phages marked at *gp38*	This study, courtesy of T.V.P. Nguyen
oTN0157	upstream-*gp38*_fwd	AGCACGTTGACCAGTCCG	construction of phages marked at *gp38*	This study, courtesy of T.V.P. Nguyen
oTN0158	upstream-*gp38*_rev	ACTGCGATGAGTGGCAGGGCGGGGCGTAATAGGATCTCCGTTTACCAGTTATAAATAATC	construction of phages marked at *gp38*	This study, courtesy of T.V.P. Nguyen
oTN0159	downstream-*gp38*_fwd	GAAAGTTGGAACCTCTTACGTGCCGATCAAAAAGCCCGCACAAGGCG	construction of phages marked at *gp38*	This study, courtesy of T.V.P. Nguyen
oTN0160	downstream-*gp38*_rev	GGACGACTATCGCGGCCA	construction of phages marked at *gp38*	This study, courtesy of T.V.P. Nguyen
oTN0161	delta*gp38*-cmR_fwdcmR_fwd	CGACGAGACGGACATGAGTT	construction of phages marked at *gp38*	This study, courtesy of T.V.P. Nguyen

oTN0162	deltagp38-cmR_rev	deltagp38-cmR_rev	construction of phages marked at *gp38*	This study, courtesy of T.V.P. Nguyen
oTN0169	*gp38*_fwd	GACGGTGAAGGGTAAGGTGG	PCR of *gp38* locus	This study, courtesy of T.V.P.Nguyen
oTN0170	*gp38*_rev	GTCCGTCCTCCTATTTGCCC	PCR of *gp38* locus	This study, courtesy of T.V.P. Nguyen
oMRS598	P*1602*_F3	TTAATACAACGAGATAATTAGTCGG	construction of pMRS70	This study
oMRS599	P*1602*_R2	GCTGGTAGCAACATTCTTCTC	construction of pMRS70	This study
oMRS630	GAB199	AGGTATGGAAGAAGGGACTCG	PCR of *gp71*-intergenic locus	This study, courtesy of G. Beggs
oMRS631	GAB200	CGTCTATTACTTCGCTATCGGATG	PCR of *gp71*-intergenic locus	This study, courtesy of G. Beggs
oMRS179	pRE112-primer2-ext	CCAATTCTGAGGGATCGGGCCCTATCACTT	construction of pBAD-q-Cm^R	This study
oMRS180	pRE112-Pcat-F	AAGGGGTGTTTCCTGGTGTCCCTGTTGATAC	construction of pBAD-q-Cm^R	This study
oMRS181	pJES-093-primer2-R	GCCCGATCCCTCAGAATTGGTTAATTGGTTGTAAC	construction of pBAD-q-Cm^R	This study
oMRS182	pJES-093-ab-F	GACACCAGGAAACACCCCTTGTATTACTGTTTATG	construction of pBAD-q-Cm^R	This study

Table S5: Plasmids used in this study

Identifier	Description	Backbone	Marker	Origin	Construction	Source
pMRS1	pRE112-*VqmA*$_{Vpara}$-ΔFLAG	pRE112	Cm	R6λγpir	Gibson assembly, SmaI site	This study
pMRS3	pTOX2-*VqmA*$_{Vpara}$-ΔFLAG	pTOX2	Cm	R6λγpir	Gibson assembly, SwaI site	This study
pMRS7	pBAD-*q*(CmR)	pEVS	Cm	p15A	Gibson assembly	This study
pMRS12	pTOX2-*VP0235*$^{+}$	pTOX2	Cm	R6λγpir	Gibson assembly, SwaI site	This study
pMRS13	pTOX2-*ΔVP0235*	pTOX2	Cm	R6λγpir	Gibson assembly, SwaI site	This study
pMRS19	pTOX2-Δ*VP0230*	pTOX2	Cm	R6λγpir	Gibson assembly, SwaI site	This study
pMRS22	pTOX2-Δ*recA*	pTOX2	Cm	R6λγpir	FastCloning, SwaI site	This study
pMRS27	pTOX2-Δ*galU*	pTOX2	Cm	R6λγpir	Gibson assembly, SwaI site	This study
pMRS28	pTOX2-Δ*nagE*	pTOX2	Cm	R6λγpir	Gibson assembly, SwaI site	This study
pMRS29	pTOX2-Δ*neuC*	pTOX2	Cm	R6λγpir	Gibson assembly, SwaI site	This study
pMRS30	pTOX2-Δ*VPA1602-1604*	pTOX2	Cm	R6λγpir	Gibson assembly, SwaI site	This study
pMRS70	pEVS-P*VPA1602*(1)-*luxCDABE*	pEVS (Ec1508)	Kan	p15A	FastCloning	This study
FJS-P073	pEVS-P*luxC*-*luxCDABE*	pEVS	Kan	p15A	X	Santoriello and Bassler 2024 (9)
pOD-51	pRE112-*luxO*$_{882}$::*luxO*$_{RIMD}$	pRE112	Cm	R6λγpir	X	Duddy et al. 2023 (1)
pJES-174	pRE112-cI$_{VP882}$, repA$_{VP882}$, *vqmA*$_{Phage}$	pRE112	Cm	R6λγpir, MJ1	X	Silpe and Bassler, 2019 (2)
pSC189	Mariner transposon delivery		Kan/ Amp	R6λγpir	X	Chiang et al. 2002(10) Obtained from the S. Helaine Lab
pMMB-tfoX-sacB	Natural transformation of *V. parahaemolyticus*	pMMB	Kan	pMMB	X	Chimalapati et al. 2018 (11)
Plasmid-Phages						
	VP882 (native)	VP882	X	RepA	WT	Lan et al. 2009 (3)
MRS-P28	VP882$^{gp38\text{::Cm}}$	VP882	Cm	RepA	Natural transformation	This study, courtesy of T.V.P. Nguyen.
MRS-P29	VP882$^{gp38\text{::Gm}}$	VP882	Gm	RepA	Natural transformation	This study
	VP882$^{gp71\text{-intergenic::Cm}}$ (Previously published as VP882$^{\text{Ctr::Cm}}$)	VP882	Cm	RepA	Natural transformation	Duddy et al. 2023 (1)
	VP882$^{\text{ctr::Tn}5}$	VP882	Cm	RepA	Natural transformation	Silpe and Bassler, 2019 (2)
MRS-P12	VP882$^{gp59(\Delta\text{P98-L120fs})}$	VP882	X	RepA	Spontaneous frameshift in cI	This study

Supplemental References:

1. Duddy OP, Silpe JE, Fei C, Bassler BL. 2023. Natural silencing of quorum-sensing activity protects *Vibrio parahaemolyticus* from lysis by an autoinducer-detecting phage. PLoS Genet 19:e1010809.
2. Silpe JE, Bassler BL. 2019. A host-produced quorum-sensing autoinducer controls a phage lysis-lysogeny decision. Cell 176:268-280.e13.
3. Lan S-F, Huang C-H, Chang C-H, Liao W-C, Lin I-H, Jian W-N, Wu Y-G, Chen S-Y, Wong H. 2009. Characterization of a new plasmid-like prophage in a pandemic *Vibrio parahaemolyticus* O3:K6 strain. Appl Environ Microbiol 75:2659–2667.
4. Makino K, Oshima K, Kurokawa K, Yokoyama K, Uda T, Tagomori K, Iijima Y, Najima M, Nakano M, Yamashita A, Kubota Y, Kimura S, Yasunaga T, Honda T, Shinagawa H, Hattori M, Iida T. 2003. Genome sequence of *Vibrio parahaemolyticus: a pathogenic* mechanism distinct from that of *V. cholerae*. The Lancet 361:743–749.
5. Ferrières L, Hémery G, Nham T, Guérout A-M, Mazel D, Beloin C, Ghigo J-M. 2010. Silent mischief: Bacteriophage Mu insertions contaminate products of <Escherichia coli</i> random mutagenesis performed using suicidal transposon delivery plasmids mobilized by broad-host-range RP4 conjugative machinery. J Bacteriol 192:6418–6427.
6. Simon R, Priefer U, Pühler A. 1983. A broad host range mobilization system for in vivo genetic engineering: Transposon mutagenesis in gram negative bacteria. Bio/Technology 1:784–791.
7. Truong TT, Vettiger A, Bernhardt TG. 2020. Cell division is antagonized by the activity of peptidoglycan endopeptidases that promote cell elongation. Mol Microbiol 114:966–978.
8. Sargen MR, Helaine S. 2024. A prophage competition element protects Salmonella from lysis. Cell Host Microbe 32:2063-2079.e8.
9. Santoriello FJ, Bassler BL. 2024. The LuxO-OpaR quorum-sensing cascade differentially controls Vibriophage VP882 lysis-lysogeny decision making in liquid and on surfaces. PLOS Genet 20:e1011243.
10. Chiang SL, Rubin EJ. 2002. Construction of a mariner -based transposon for epitope-tagging and genomic targeting. Gene 296:179–185.
11. Chimalapati S, De Souza Santos M, Servage K, De Nisco NJ, Dalia AB, Orth K. 2018. Natural Transformation in *Vibrio parahaemolyticus* : a Rapid Method To Create Genetic Deletions. J Bacteriol 200.
